# Successful Treatment of Fetal Intraperitoneal Administration of Immunoglobulin in a Case of Fetal Hemolytic Anemia with 131,072-Fold Anti-E Alloimmunization

**DOI:** 10.1155/2011/157510

**Published:** 2011-11-17

**Authors:** Masashi Yoshida, Hideo Matsuda, Eijiro Hayata, Akio Watanabe, Miho Oeda, Kenichi Furuya

**Affiliations:** ^1^Department of Obstetrics and Gynecology, National Defense Medical College, 3-2 Namiki Tokorozawa, Saitama 3598513, Japan; ^2^Division of Obstetrics and Gynecology, Matsuda Perinatal Clinic, 1080-5 Hongo, Tokorozawa, Saitama 3590022, Japan

## Abstract

*Object*. We present here the case of severe fetal anemia caused by anti-E antibody positive, which showed a favorable course only with fetal intraperitoneal administration of immunoglobulin. *Case*. The mother was 31 years old, blood type B Rh : CCDee, gravida 1, with no history of transfusion. Anti-E antibody was detected in the maternal cross-match test at the 18th gestational week. In percutaneous umbilical blood sampling, the umbilical blood hemoglobin level was 9.1 g/dL and the result of the direct Coombs' test was positive at the 26th gestational week. Immunoglobulin injection into fetal abdominal cavity (IFAC) was administered a total 7 times. During the pregnancy, the indirect Coombs' test showed a 131,072-fold increase. *Conclusion*. In this case, IFAC to block the reticuloendothelial system Fc receptor was successful. This procedure is promising as one of the treatment options for blood group incompatible pregnancy.

## 1. Introduction

Irregular antibodies are usually detected in 0.3% of all pregnancies [1]. The management of Rh-D blood type incompatible pregnancy is endorsed by the American College of Obstetricians and Gynecologists (ACOG) [2, 3]. The established treatment for prevention of sensitization during pregnancy is maternal administration of anti-D immunoglobulin [4, 5]. However, the application of this treatment for other blood-type incompatible pregnancies has not been clearly indicated. In general, fetal transfusion is recommended when anemia is confirmed by ultrasonography or percutaneous umbilical blood sampling. In the present case, we administered immunoglobulin injection into fetal abdominal cavity (IFAC) with severe anemia caused by anti-E antibody. Following administration, the anemia improved and the fetus showed a favorable postnatal course.

## 2. Case

The patient was a 31-year-old female, B Rh : CCDee, gravida 1, parity 1, height 171 cm, prepregnancy weight 72 kg, prepregnancy BMI 24.6, with no history of blood transfusion. The father's blood type was B Rh : ccDEE. 

Anti-E antibody was detected at the early period of pregnancy, and the patient was referred to our hospital at the 18th gestational week. The result of the indirect Coombs' test was 1 : 2,048, and a high antibody titer was maintained, as shown in subsequent indirect follow-up Coombs' tests performed every 4 weeks. Although fetal hydrops did not develop, middle cerebral artery peak systolic velocity was acutely elevated to 45.4 cm/s at the 26th gestational week, suggesting progressive anemia.

The patient was immediately admitted and fetal anemia was evaluated by percutaneous umbilical blood sampling (PUBS). The umbilical venous hemoglobin level was 9.1 g/dL, and the hematocrit level was 26.5%. The fetal blood type was B Rh : CcDEEe, and the indirect Coombs' test was positive. On the same day, immunoglobulin was administered intraperitoneally to the fetus at a dose of 2 g/kg estimated body weight using a 25-gauge needle (Hanaco Medical, Saitama, Japan). Diazepam 10 mg, pentazocine 30 mg, and ketamine 30 mg were used for fetal anaesthesia. More treatment sessions of IFAC were performed for a total of 7 times. Fetal transfusion was not carried out at this time. During IFAC treatment, the indirect Coombs' test value increased, transiently reaching 131,072-fold.  Table 1 shows the hematologic profile before and after IFAC. The nonstress test showed a reassuring fetal status pattern during treatment.

The mother delivered a male baby weighing 3,430 g by planned transvaginal delivery at 36 weeks. The Apgar scores were 6/7, 1-minute/5-minutes, respectively. The hemoglobin level was 13.6 g/dL and the hematocrit level was 43.1% immediately after birth. The indirect Coombs' test was positive. Postpartum, preventative phototherapy was carried out, but at 2 days exchange transfusion was required (total-bilirubin 16.2 mg/dL, LDH 590IU/L, hemoglobin 12.7 g/dL, hematocrit 40.3%).

 The baby was discharged on the 22th day. No signs of anemia or developmental delay were identified until 18 months after birth.

## 3. Discussion

We previously reported the other case of fetal hemolytic anemia with anti-M alloimmunization [6]. However, the standard injection methods of immunoglobulin to fetuses have not yet been established. In general, fetal transfusion is required for the treatment of fetal anemia in blood group incompatible pregnancy. Maternal immunoglobulin administration has recently been suggested for the treatment of blood type incompatible pregnancy to replace invasive forms of treatment such as plasma exchange or fetal transfusion. There have been reports that anti-D antibody titer was stabilized 6-7 weeks after maternal immunoglobulin administration and plasma exchange [7]. However, there is as yet no report on the favorable prognosis in newborns only with maternal immunoglobulin administration. A previous report has described a patient with Rh-D incompatible pregnancy continuously administered with maternal immunoglobulin at a dose of 1 g/kg maternal body weight from the 14th gestational week. Fetal transfusion was performed concurrently from the 20th gestational week, and the baby was delivered at the 34th gestational week [8]. In another patient, although immunoglobulin administration was started from the 10th gestational week, fetal transfusion was technically difficult and was performed only once in the third trimester before birth [9]. Chitkara et al. reported that immunoglobulin therapy was ineffective in 4 patients with Rh-D incompatible pregnancy [10]. However, there are few negative reports on immunoglobulin therapy at present.

The effects of immunoglobulin administration in blood group incompatible pregnancy have not been fully clarified to date. However, the following effects were suggested: (1) prevent antibody combined cell destruction by blocking Fc receptors in the reticuloendothelial system, (2) act as a neutralizing antibody, (3) aid in the deposition of the immune complex, (4) increase the number of suppressor T-cells, and (5) decrease the number of killer cells and peripheral lymphocytes [11–13]. Maternal immunoglobulin administration has an additional effect in preventing the placental transfer of irregular antibodies [14]. Furthermore, *in vitro* studies have proven that immunoglobulin administration prevents placental transfer of a pathogenic antibody [15]. However, neutralization of an antibody transferred to the fetus cannot be achieved by maternal immunoglobulin administration.

Postnatal immunoglobulin therapy has been reportedly used for the treatment of blood type incompatible pregnancy. There has been a report on a patient treated with 0.5 g/kg of immunoglobulin added to 20 mL/kg of packed RBC [16]. Although fetal transfusion is usually possible at the 22nd gestational week, it is technically challenging and incurs the risk of developing bradycardia. Furthermore, the safety of blood preparations and changes in the potassium ion should be considered. On the other hand, the advantages of IFAC include lower cost than RBC preparations, lower risk of infection because of the heating process, and no risk of developing hemochromatosis. 

 From this background, we devised the immunoglobulins administration in the fetal abdominal cavity. Although there is a chance of puncturing the umbilical cord during IFAC, having obtained a live birth safely in the present case through the direct administration of immunoglobulin to the fetus without the need for intrauterine blood transfusion, even when the indirect Coombs' test antibody titer had increased as high as 131,072-fold, can be regarded as highly significant.

 The other methods for fetal immunoglobulin include umbilical venous administration. Although umbilical venous administration is physiologically feasible, it carries the risk of developing bradycardia from umbilical artery puncture, aside from requiring expertise in the procedure. On the other hand, intraabdominal administration creates a broader puncture area technically. Since intraabdominal administered immunoglobulin is readily absorbed through subdiaphragmatic lymphatics, sufficient effect can be expected. We reported that immunoglobulins are incorporated into the fetal circulation following IFAC [17]. The improved safety of fetal treatment using 25-gauge needles has been reported by Kawakami et al. [18]. Here, we used a high dose of immunoglobulin (2 g/kg) for the fetus, which is within the therapeutic range for the Kawasaki disease [19]. The administration of 2 g/kg of immunoglobulin into the fetal abdominal cavity did not induce symptoms of heart failure from volume overload, and complete immunoglobulin absorption occurred after 3 days. Although the used dose is not considered an overdose presently, additional studies are required to determine the adequate dosage.

## 4. Conclusion

In this case, IFAC to block the reticuloendothelial system Fc receptor was successful. This procedure is promising as one of the treatment options for blood group incompatible pregnancy considering the merit of a heat-treated product.

## Figures and Tables

**Table 1:** The clinical course.

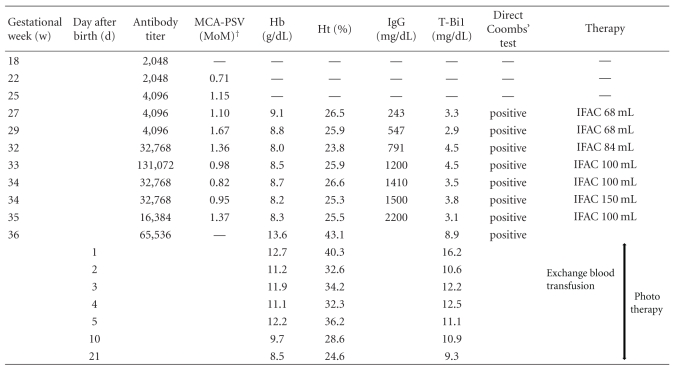

MCA-PSV (MoM)^†^: middle cerebral artery-peek systolic velocity (multiple of median).
